# Safety, biodistribution and radiation dosimetry of [^123^I]ioflupane in healthy Chinese volunteers

**DOI:** 10.1186/s13550-023-00978-3

**Published:** 2023-04-07

**Authors:** Min Zhang, Yue Wang, Jin Wang, Xiang Li, Biao Li

**Affiliations:** Department of Nuclear Medicine, Ruijin Hospital, Shanghai Jiao Tong University School of Medicine, 197 Ruijin 2nd Road, Shanghai, 200025 China

**Keywords:** [^123^I]Ioflupane, Biodistribution, Radiation dosimetry, SPECT, Chinese volunteers

## Abstract

**Background:**

[^123^I]ioflupane is a radiopharmaceutical used to visualise the dopaminergic neuron terminals in the striata, to aid in the differential diagnosis among Parkinsonian syndromes (e.g., Parkinson’s disease). However, nearly all of the subjects in the initial development studies of [^123^I]ioflupane were Caucasian.

**Methods:**

8 Chinese healthy volunteers (HVs) received a single 111 MBq ± 10% dose of [^123^I]ioflupane and had simultaneous whole-body (head to mid-thigh) anterior and posterior planar scintigraphy scans at 10 min and 1, 2, 4, 5, 24, and 48 h. To estimate biodistribution, dosimetry was evaluated for the Cristy–Eckerman female and hermaphrodite male phantoms. Single-photon emission computed tomography (SPECT) images of the brain were acquired at 3 and 6 h after injection. Blood samples and all voided urine were collected for 48 h for pharmacokinetic analysis. The results were then compared with those of a similar European study.

**Results:**

There were strong similarities in uptake and biodistribution between the Chinese and European studies. Excretion was primarily renal, and the values were similar for the first 5 h but diverged after that, possibly because of differences in subjects’ height and weight. Tracer uptake in regions of interest in the brain was stable over the imaging window of 3 to 6 h. The difference in mean effective dose for Chinese HVs vs European HVs (0.028 ± 0.00448 vs 0.023 ± 0.00152 mSv/MBq) was not clinically significant. The [^123^I]ioflupane was well tolerated**.**

**Conclusion:**

This study demonstrated that a single 111 MBq ± 10% dose of [^123^I]ioflupane injection was safe and well tolerated, and the SPECT imaging window of 3 to 6 h after injection of [^123^I]ioflupane was appropriate in Chinese subjects.

*Trial registration number* ClinicalTrials.gov: NCT04564092.

## Introduction

Ioflupane is a cocaine analogue (tropane derivative) that binds reversibly with high affinity to the dopamine transporter (DaT or DAT), a marker for presynaptic terminals in dopaminergic nigrostriatal neurons [1–4]. When labelled with radioactive iodine 123, it can be used for single-photon emission tomography (SPECT) imaging of the dopaminergic tracts in the striata. [^123^I]ioflupane has been marketed (as DaTscan™ in the United States and as DaTSCAN™ in other countries) for more than 10 years to assist in the evaluation of adult patients with suspected Parkinsonian syndromes (PS). In these patients, DaTscan™ may be used to help differentiate essential tremor from tremor due to Parkinsonian syndrome (PS), which includes idiopathic Parkinson’s disease (PD), multiple system atrophy (MSA), and progressive supranuclear palsy (PSP) [5, 6]. However, 99% of the subjects who took part in the registration studies of DaTscan™ were Caucasian [7]. The present phase I study prospectively evaluates the biodistribution, radiation dosimetry, and effective dose of [^123^I]ioflupane in healthy Chinese volunteers and compares the values to those from a 1998 phase 1 study of European volunteers [8].

## Methods

### Subjects

This study sponsored by GE Healthcare (ClinicalTrials.gov identifier: NCT04564092) was conducted according to International Conference on Harmonisation (ICH) Good Clinical Practice Guideline. It was approved by the Ethics committee of the Ruijin hospital. This single-centre study recruited healthy Chinese volunteers. Eligible subjects were generally healthy and fit Chinese males or females aged 18 to 70 years and with a body mass index (BMI) of 18 to 30 kg/m^2^. Pregnant or lactating women were ineligible, and women of childbearing potential had to be willing to use effective contraception. Subjects would be excluded if they had a history of motor disturbances; a history of pulmonary, cardiovascular, renal, hepatic, coagulation, or hormonal disorders, including hyperthyroidism; a history of drug, alcohol, or solvent abuse; use of any investigational medicinal product (IMP) within 30 days prior to screening or receipt of any radionuclide injection within a minimum of 5 radioactive half-lives prior to screening; use of any medication except acetaminophen (paracetamol) or oral contraceptive, including traditional Chinese medications, within 2 weeks of the imaging visit; or classification as a radiation worker.

Subjects were to attend 3 visits: screening, imaging, and follow-up. Medical history, physical examination, clinical laboratory tests and urine analysis, and 12-lead Electrocardiogram (ECG) were obtained for screening and safety assessment purposes. To minimise thyroid uptake of radioactive iodine, subjects had to have appropriate thyroid blocking, according to local practice, prior to and after administration of the IMP.

### objective

The primary objective of this phase 1 study was to evaluate the safety of a single dose of [^123^I]ioflupane injection in Chinese healthy volunteers (HVs). The secondary objectives were: (1) To determine the biodistribution, internal radiation dosimetry, and effective dose (ED) of [^123^I]ioflupane injection after intravenous (IV) administration in Chinese HVs. (2) To compare biodistribution and dosimetry findings in this Chinese group with those previously established in European HVs. (3) To compare the brain SPECT imaging results in this Chinese group with those previously established in European HVs.

### Investigational medicinal product (IMP)

GE Healthcare was responsible for the manufacturing, distribution, and reconciliation of the IMP, [^123^I]ioflupane injection. The IMP was prepared at GE Healthcare BV, Eindhoven, Netherlands.

### Image acquisition

Whenever possible, images were acquired at prespecified time points. During the imaging visit, each subject received a single IV bolus injection of [^123^I]ioflupane with a nominal ^123^I activity of 111 MBq ± 10% and underwent simultaneous whole-body (head to toe) anterior and posterior planar scintigraphy scans on Intevo 16 SPECT/CT system (Siemens, Erlangen, Germany) at 10 min, 1 h, 2 h, 4 h, 5 h, 24 h, and 48 h after administration. Attenuation correction was performed using transmission scans acquired using a flood source filled with a solution of ^123^I. Brain SPECT imaging was performed at 3 and 6 h after administration. A reference source was imaged alongside the subject. Whole-body planar images were acquired in a 256 × 1024 matrix and a low-energy, high-resolution, parallel-hole collimator. Brain SPECT acquisition was performed using the same collimator, a 128 × 128 matrix, and a total of 32 frames over 180° with 30 s per frame. After acquisition, Butter-worth low-pass filter and Ramp function were used to reconstruct transverse images.

### Blood collections

Blood samples were collected before the [^123^I]ioflupane injection and at 5, 15, 30 min and 1, 2, 3, 4, 5, 24, and 48 h after injection for pharmacokinetic analysis. For each HV, up to 5-mL venous blood samples were taken at each time point to allow measurement of ^123^I content in whole blood and plasma over time. The maximum amount of blood taken for activity counting was 55 mL for each HV.

### Urine collections

Urine excreted from 1 h before administration of [^123^I]ioflupane injection to 48 h after injection was collected as voided. The time and volume of each void was recorded. From each void, 3 aliquots of urine of a nominal 1 mL volume each were taken and assayed for ^123^I activity content over 60 s intervals.

### Data analysis

The striatal binding ratio (SBR) is a measure of how much [^123^I]ioflupane binds to DaT in different regions of the brain compared to a reference region where there is no binding. DaTQUANT™ software was used to automatically delineate the volumes of interest (VOIs) in the striatal regions and reference region (occipital lobe), and quantify left and right SBRs from SPECT images acquired at approximately 3 and 6 h post-injection for each subject. The VOIs of sub-regions over the putamen and caudate were also considered using DaTQUANT™ software. To calculate the SBR, DaTQUANT™ uses a formula that involves dividing the average pixel intensity of a VOI by the average pixel intensity of a reference region (REF). The formula is: SBR = (VOI − REF)/REF.

### Radiation dose calculation

Time-activity curves were then generated from attenuation corrected, conjugate view ^123^I activity data for each subject and parameterised by mono- or biexponential curve fitting to calculate the normalised cumulative activity in each source region using the OLINDA/EXM Version 1.1 software [9], which was then used along with the Medical Internal Radiation Dosimetry (MIRD) schema to determine the internal radiation dosimetry [10]. The dosimetry was evaluated for the Cristy–Eckerman female and hermaphrodite male phantoms [11].

### Statistical analysis

Tabulations of summary statistics, graphical presentations, and statistical analyses were performed by using SAS® software, Version 9.4. The last observation prior to administration of IMP was used as the baseline value for calculating post-administration changes from baseline. *p*-values was interpreted as a metric of uncertainty. Confidence intervals, both individual and simultaneous, were at the 95% confidence level. The 2-sample *t*-test was used to compare the radiation dosimetry data from the Chinese healthy volunteers (HVs) in this study to the HVs in the earlier European study [8]. The difference between the SBR at 3 and 6 h calculated for each subject was tested by Wilcoxon signed-rank test. No sample size calculation was performed. The sample size was chosen to fulfil regulatory requirements.

## Results

### Study population

The study was initiated on 14 May 2021 and completed on 04 September 2021. All 8 of the enrolled subjects who received [^123^I]ioflupane completed the study and were included in the pharmacokinetic population.

All subjects were Chinese; the majority were Han Chinese (6 subjects,75%). Four (50%) were male, and 4 (50%) were female. Age ranged from 24 to 41 years, with a median of 29.0 years. Mean height, weight, and body mass index of the subjects were 164.69 cm, 58.18 kg, and 21.099 kg/m^2^, respectively.

All subjects received thyroid blocking medication. All subjects received a dose of [^123^I]ioflupane in the planned range of 111 MBq ± 10%. The mean (SD) dose of radioactivity injected was 114.6 (5.15) MBq.

### Biodistribution

Blood sampling was performed for all 8 subjects. Table 1 summarizes the biodistribution of [^123^I]ioflupane, expressed as the decay-corrected percentage of injected activity in plasma and whole blood. Blood clearance of ^123^I activity was shown to be rapid. At 5 min after injection, the percentage of injected activity (%IA) present within the blood was 4.564 ± 1.471, falling to 2.220 ± 0.504 at 30 min. The %IA in whole blood was maintained for up to 5 h after injection (1.924 ± 0.403) and showed a gradual reduction to 1.348 ± 0.339 at 24 h and 1.098 ± 0.344 at 48 h after injection. The %IA in plasma was 2.769 ± 0.773 at 5 min, 1.350 ± 0.293 at 30 min, 0.768 ± 0.181 at 24 h, and 0.626 ± 0.138 at 48 h after injection. The %IA values were similar for both whole blood and plasma.

**Table 1:** Summary of biodistribution, decay-corrected percentage injected activity (%IA) in blood, PK population (N = 8)

		Time after dosing									
Tissue	Predose	5 min	15 min	30 min	1 h	2 h	3 h	4 h	5 h	24 h	48 h
Plasma											
Mean (SD)	0.016 (0.007)	2.769 (0.773)	1.686 (0.557)	1.350 (0.293)	1.351 (0.267)	1.375 (0.340)	1.338 (0.385)	1.229 (0.336)	1.138 (0.300)	0.768 (0.181)	0.626 (0.138)
Whole blood											
Mean (SD)	0.030 (0.009)	4.564 (1.471)	2.798 (0.941)	2.220 (0.504)	2.220 (0.472)	2.355 (0.550)	2.216 (0.550)	2.081 (0.492)	1.924 (0.403)	1.348 (0.339)	1.098 (0.344)

*SD* Standard deviation

**Fig. 1:**
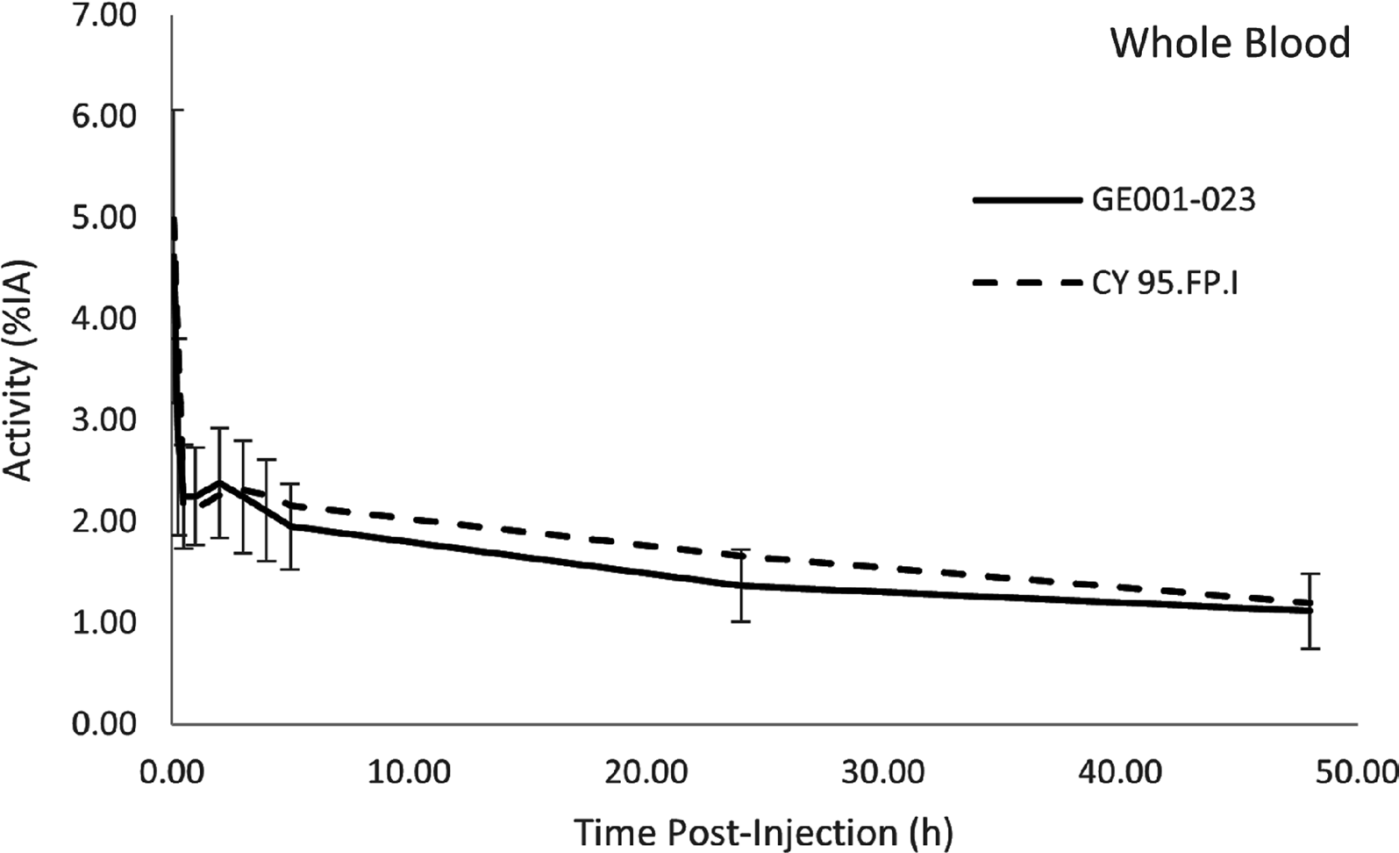
Mean whole blood.^123^I activity curves in Chinese HVs (solid line, GE001-023, error bars represent one standard deviation.) vs the HVs in a European phase 1 study (dashed line, CY95.FP.1) [8]

Figure 1 compares the mean whole blood activity curves of [^123^I]ioflupane in this study (GE001-023) with those obtained during the earlier phase I study (CY 95.FP.1) [8] that enrolled only Europeans (Caucasians). There was no significant difference in decay-corrected %IA in mean whole blood between Chinese and European subjects at any time point.

**Fig. 2:**
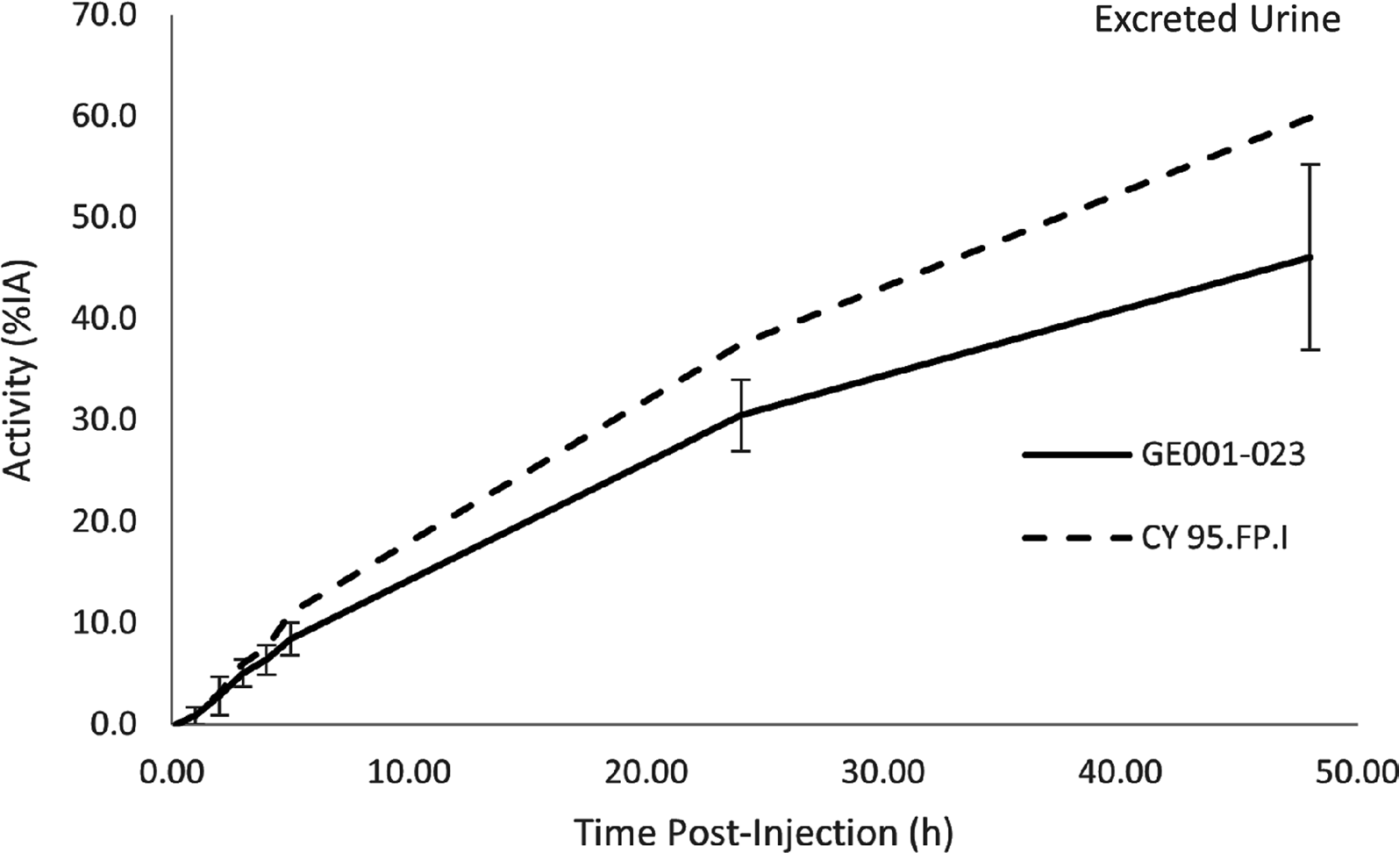
Mean excreted urine ^123^I activity curves in Chinese HVs (solid line, GE001-023, with error bars at one standard deviation) vs the HVs in a European phase 1 study (dashed line, CY95.FP.1) [8]. %*IA* percentage of injected activity; *h* hours

Figure 2 shows the mean cumulative, decay-corrected and normalised ^123^I activity excreted in urine for all 8 Chinese subjects where samples were acquired together with the mean curve from the 12 subjects in the European study [8]. Excretion of ^123^I following the IV administration of ioflupane (^123^I) injection was determined by measuring the ^123^I activity within the intestinal contents and the sum of the ^123^I activities in the voided urine up to 48 h post-injection. During the period of 0 to 5 h after injection, the cumulative mean activity excreted in urine was 8.37 ± 1.73%IA. The mean %IA continued to increase over the period from 5 to 48 h after injection. By 48 h after injection, the mean sum of activity in voided urine was 45.45 ± 8.73% (range: 29.10% to 55.90%) of that injected. If that value is extrapolated for infinite time, the total activity excreted via the renal pathway is 57.40% (range: 31.30% to 89.40%). There was no statistically significant difference in decay-corrected cumulative ^123^I activity concentration in excreted urine between Chinese and European subjects at time points up to 5 h. A significant difference was seen at 5 to 24 h (*p* = 0.0204) and 24 to 48 h (*p* = 0.0029).

Table 2 summarizes the biodistribution in Chinese subjects. The source regions with the highest decay-corrected normalised cumulative activities were remainder (9.409 MBq·h/MBq), lungs (1.664 MBq·h/MBq), and liver (1.255 MBq·h/MBq). Table 3 shows that there was a strong similarity in biodistribution in terms of the decay-corrected normalised cumulative activity between Chinese and European subjects. The 3 source regions with the highest mean normalised cumulative activity were the same in the Chinese HVs and the subjects from the European study, and there was no significant difference between Chinese and European subjects.

**Table 2:** Summary of biodistribution, decay-corrected normalised cumulative activity (MBq·h/MBq)

Tissue	Pharmacokinetic population [Mean (SD)] (N = 8)
Bladder contents	0.548 (0.076)
Brain	0.659 (0.154)
Gallbladder	0.046 (0.018)
Liver	1.255 (0.468)
Lower large intestine	0.460 (0.163)
Lungs	1.664 (0.268)
Remainder	9.409 (1.042)
Small intestine	0.290 (0.103)
Upper large intestine	0.562 (0.199)

*SD* standard deviation

**Table 3:** Biodistribution in Chinese HVs versus HVs in a European Study [8]

Tissue	Source regions with the 3 highest mean normalised cumulative activities (MBq·h/MBq)		
	Chinese (N = 8)	European (N = 12)	*p*-value
	Mean (SD)	Mean (SD)	
Remainder	9.409 (1.042)	9.340 (1.200)	0.8965
Lungs	1.664 (0.268)	1.480 (0.515)	0.3680
Liver	1.255 (0.468)	1.120 (0.208)	0.3876

*HV* healthy volunteer

### Striatal binding ratio (SBR)

**Fig. 3:**
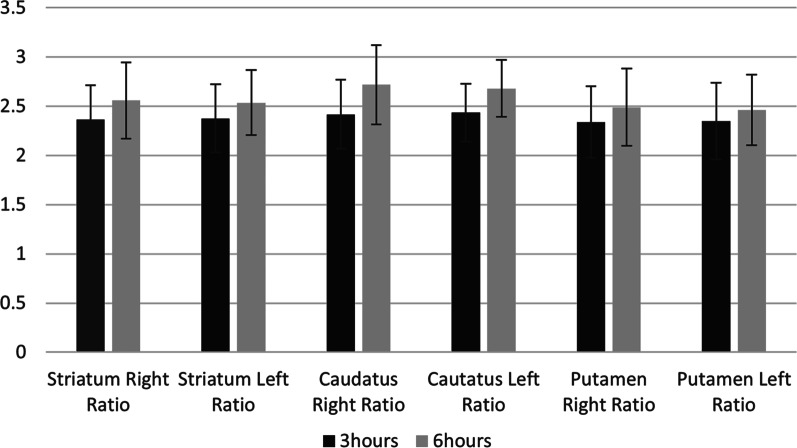
Analysis of striatal binding ratio (SBR) at 3 and 6 h after administration of [^123^I]ioflupane

Mean SBR in the right striatum was 2.362 at 3 h after injection and 2.558 at 6 h after injection. Mean SBR in the left striatum was 2.376 at 3 h after injection and 2.535 at 6 h after injection. There was no significant difference in SBR at 3 and 6 h post-administration in the striatum, putamen, and caudate (Fig. 3).

### Dosimetry

Table 4 summarizes radiation dosimetry in Chinese HVs. The effective dose (ED) in the Chinese HVs in this study and the HVs in the earlier European study [8] was compared. The mean ED calculated in this study of Chinese HVs (0.028 ± 0.00448 mSv/MBq) while the sex-averaged ED was 0.027 mSV/MBq (0.025 mSv/MBq male average, 0.032 mSv/MBq female average), this was similar to the value calculated in the study of European HVs (0.023 ± 0.00152 mSv/MBq), the sex-average effective dose determined here is aligned with the published value of 0.025 mSV/MBq for nortropane (FP-CIT) [12].

**Table 4:** Absorbed effective dose in Chinese HVs

Target organ	Absorbed dose (mGy/MBq) ± SD
Urinary bladder wall	0.060 ± 0.01423
LLI wall	0.052 ± 0.01653
Lungs	0.052 ± 0.00706
ULI wall	0.048 ± 0.01548
Liver	0.035 ± 0.00838
Bone surfaces (osteogenic cells)	0.033 ± 0.00504
Gallbladder wall	0.030 ± 0.00540
Small intestine	0.026 ± 0.00675
Heart wall	0.024 ± 0.00478
Brain	0.021 ± 0.00573
Stomach	0.021 ± 0.00439
Ovaries	0.020 ± 0.00451
Uterus	0.018 ± 0.00281
Spleen	0.018 ± 0.00550
Pancreas	0.016 ± 0.00194
Thyroid	0.016 ± 0.00471
Adrenals	0.015 ± 0.00161
Kidneys	0.013 ± 0.00150
Thymus	0.012 ± 0.00149
Muscle	0.011 ± 0.00138
Red marrow	0.010 ± 0.00129
Breasts	0.009 ± 0.00098
Testes	0.008 ± 0.00091
Skin	0.006 ± 0.00086
Total body	0.013 ± 0.00162
Effective dose (mSv/MBq)	0.028 ± 0.00448

*LLI* lower large intestine; *ULI* upper large intestine

There was a strong similarity between radiation dosimetry in Chinese and European subjects. The 3 target organs (urinary bladder wall, lower large intestine wall, lungs) with the highest mean absorbed doses as well as the mean absorbed doses in the brain were the same in the Chinese HVs and the European subjects, and there was no significant difference between Chinese and European subjects (Table 5).

**Table 5:** Summary of radiation dosimetry in Chinese HVs vs HVs in a European Study [8]

Target organ and tissue	Target organs and tissues including 3 highest mean absorbed doses (mGy/MBq)						
	Chinese (,N = 8)			European (N = 12)			*p*-value
	Mean	Min	Max	Mean	Min	Max	
Urinary bladder wall	0.060	0.042	0.082	0.053	NA	NA	0.1981
LLI wall	0.052	0.027	0.084	0.042	NA	NA	0.0537
Lungs	0.052	0.044	0.060	0.042	NA	NA	0.0617
Brain	0.021	0.016	0.027	0.018	NA	NA	0.1599

*LLI* lower large intestine; *max* maximum; *min* minimum; *NA* Not available; *ULI* upper large intestine. *N* Total number of subjects in the pharmacokinetic population for that cohort

### Safety assessment

Safety analysis included adverse events (AEs), injection-site monitoring, physical examination, vital signs and oxygen saturation, clinical laboratory tests, ECG etc. In this study of Chinese HVs, [^123^I]ioflupane injection was well tolerated. There were no deaths or other serious adverse events (SAEs), and no AEs led to discontinuation of the study. The majority of AEs were mild in intensity, the most common AEs reported was paraesthesia.

## Discussion

This was the first study to evaluate the safety, biodistribution, internal radiation dosimetry, and ED of a single 111 MBq ± 10% dose of [^123^I]ioflupane injection in Chinese HVs. The primary objective of the study was to evaluate safety. All parameters assessed during this study demonstrated that a single 111 MBq ± 10% dose of [^123^I]ioflupane injection was safe and well tolerated.

Apart from the good safety profile, the results were strongly consistent with those of an earlier European study [8].

There was a strong similarity in the biodistribution of ^123^I activity in these Chinese subjects as compared to the European subjects in the earlier study. Blood clearance of ^123^I activity was shown to be rapid, decreasing from 4.564 ± 1.471%IA in whole blood at 5 min after injection to 2.220 ± 0.504%IA at 30 min after injection. Similar values were seen in the European study.

Excretion of ^123^I activity in Chinese subjects was primarily renal, with approximately 45% of the dose (calculated as cumulative decay-corrected ^123^I activity in collected urine) excreted within 48 h after injection. At timepoints up to 5 h after administration, there was no statistically significant difference in the urinary excretion of [^123^I]ioflupane (decay-corrected cumulative ^123^I activity concentration in excreted urine) between the Chinese HVs in this study and the European HVs in the earlier study. After 5 h, the curves diverged slightly, suggesting slower renal excretion among the Chinese cohort (45.5% vs 59.9% of the administered dose had been excreted by 48 h after administration). There were significant differences between the Chinese and European cohorts for the cumulative urinary excretion from 5 to 24 h and 24 to 48 h. However, these differences may be due to differences in subjects’ height and weight (the Chinese subjects were generally smaller). Renal excretion rate has been shown to depend on body size [13, 14].

Radiation dosimetry was similar in Chinese and European subjects. The 3 target organs/tissues with the highest mean absorbed doses were the same in the Chinese HVs and the European HVs, and there were no statistically significant differences between Chinese and European HVs in the mean absorbed doses in these organs/tissues. These organs/tissues were the wall of the urinary bladder (0.060 mGy/MBq for Chinese subjects vs 0.535 mGy/MBq for Europeans), the wall of the lower large intestine (0.042 vs 0.064 mGy/MBq), and the lungs (0.050 vs 0.055 mGy/MBq).

The mean normalised ED for all subjects in the Chinese study was 0.028 ± 0.00448 mSv/MBq. while the sex-averaged ED was 0.027 mSV/MBq (0.025 mSv/MBq male average, 0.032 mSv/MBq female average). The sex-average effective dose determined here is aligned with the published value of 0.025 mSv/MBq for nortropane [12]. This was also similar to the value derived from the European study (0.023 ± 0.00152 mSv/MBq). Although there was a good agreement between sex-averaged absorbed doses in Chinese and European HVs, dose estimates in the Chinese HVs were slightly, but consistently, higher than those in the European HVs. Given the strong similarity in the biodistribution observed in these 2 studies, this bias may be attributable to differences between software tools used for the modelling of residence times. The difference in urinary excretion rate may also be a factor. However, the difference in ED between Chinese and European subjects remains very small with no clinical significance.

In this Chinese cohort, the mean SBR in the right striatum was 2.362 at 3 h after injection and 2.558 at 6 h after injection. The mean SBR in the left striatum was 2.376 at 3 h after injection and 2.535 at 6 h after injection. There was no significant difference in SBR at 3 vs 6 h post-administration in the striatum, putamen, and caudate. These findings confirm the stability of tracer uptake in these regions relative to a reference region (occipital lobe) over the proposed clinical imaging window of 3 to 6 h [15]. Thus, the imaging window is appropriate for Chinese subjects, as it is for European subjects.

In addition, we notice that DAT PET (^18^F-FP-CIT) has been used in multiple Chinese PET centres. Although its image quality may be better than DAT SPECT, a previous study has demonstrated a high concordance of visual assessment results between DAT SPECT and DAT PET, and a significant correlation between the SBR measured by the two different tracers [16]. Considering the advantages of SPECT over PET, including cyclotron-independence, lower examination price, and more widely available equipment, DAT SPECT is still a valuable imaging tool for the detection of striatal dopaminergic degeneration in China.

As a conclusion, this study demonstrated that a single 111 MBq ± 10% dose of [^123^I]ioflupane injection was safe and well tolerated in Chinese subjects. It also confirmed that the brain SPECT imaging window of 3 to 6 h after injection of [^123^I]ioflupane was appropriate for imaging striata.

### Limitations

First, this study used standard methods for radiation dosimetry. Differences in the software tools used for modelling residence times could explain small, clinically insignificant differences in dose estimates between the European and Chinese populations. Second, we used the data measured by whole-body planar imaging to calculate initial organ uptake, normalized cumulative activity, absorbed dose, and effective dose. Although the overlap of tissues or organs in planar imaging may make the accuracy of delineating the region of interest limited, planar imaging is still a feasible way to assess the dosimetry of tissues and organs throughout the body.

## Data Availability

The datasets generated during and/or analysed during the current study are available from the corresponding author on reasonable request.
